# Characterization of acidic lysine acylations in mycobacteria

**DOI:** 10.3389/fmicb.2024.1503184

**Published:** 2024-12-10

**Authors:** Tong Ye, Danfeng Wang, Yewen Sun, Shuyu Xie, Tianqi Liu, Nana Tian, Minjia Tan, Jun-Yu Xu

**Affiliations:** ^1^School of Chinese Materia Medica, Nanjing University of Chinese Medicine, Nanjing, Jiangsu, China; ^2^State Key Laboratory of Drug Research, Shanghai Institute of Materia Medica, Chinese Academy of Sciences, Shanghai, China; ^3^School of Pharmacy, Zunyi Medical University, Zhuhai, China; ^4^Zhongshan Institute for Drug Discovery, Shanghai Institute of Materia Medica, Chinese Academy of Sciences, Zhongshan, Guangdong, China

**Keywords:** *Mycobacterium smegmatis*, acidic lysine acylation, lysine malonylation, lysine succinylation, lysine glutarylation, functional regulation

## Abstract

**Introduction:**

Protein acetylation is an extensively investigated post-translational modification (PTM). In addition to lysine acetylation, three new types of lysine acylations characterized by the presence of an acidic carboxylic group have been recently identified and validated. These included lysine malonylation (Kmal), lysine succinylation (Ksucc) and lysine glutarylation (Kglu). Pathogens belonging to the genus Mycobacterium elicit severe diseases in mammalian hosts through the modulation of energy metabolism pathways. Throughout this process, malonyl-CoA, succinyl-CoA and glutaryl-CoA are important intermediates in metabolic pathways, including the tricarboxylic acid (TCA) cycle, amino acid and lipid metabolism. These short-chain acyl-CoAs serve as substrates for corresponding acidic lysine acylation reactions. However, the landscape of these acyl-CoAs dependent acidic lysine acylomes remains unclear.

**Methods:**

We used the high-affinity antibody enrichment combined with high-resolution LC-MS/MS analysis to systematically investigate the global proteomic characteristics of the three acidic lysine acylations in *Mycobacterium smegmatis*. Subsequently, we employed in vitro enzymatic assays to validate the functional impact of acylated substrates, adenylate kinase and proteasome-associated ATPase. Furthermore, we investigated the effects of overexpressing these two substrates on the in vitro growth of *Mycobacterium smegmatis*, its invasion of THP-1 cells, and the influence on inflammatory cytokines.

**Results:**

We systematically investigated the global substrate characterization of 1,703 lysine malonylated sites, 5,320 lysine succinylated sites and 269 lysine glutarylated sites in the non-pathogenic model strain *Mycobacterium smegmatis*. Bioinformatics analysis demonstrated a correlation between these acidic lysine acylations and the functional roles of ribosomes, in addition to their roles in various metabolic pathways. Furthermore, we investigated the impact of lysine acylations on the functional activity of adenylate kinase and proteasome-associated ATPase, as well as their roles in mycobacterial infection process.

**Discussion:**

Collectively, our study provided an important resource on substrate characterization and functional regulation of acidic lysine acylations in *Mycobacterium smegmatis*, giving valuable insights into their interrelation with the biology of infectious process.

## 1 Introduction

Approximately 50% of the amino acids are post-translational modified, with variations ranging from simple chemical groups such as methyl to more intricate structures such as poly-peptide chains (Olsen and Mann, 2013). Protein post-translational modifications (PTMs) have the ability to alter the chemical structures of the modified residues, consequently affecting the charge, binding affinity and then biological properties of proteins (Vu et al., 2018). In comparison with tens of thousands of protein modification sites identified in eukaryotic organisms, PTMs in bacteria exhibited lower abundance and greater diversity, highlighting their unique regulatory roles (Macek et al., 2019).

Among them, lysine acetylation in bacteria results in an enlargement of the side chain and neutralization of the positive charge of amino group, which was enzymatically catalyzed with acetyl-coenzyme A (Ac-CoA) and acetyl phosphate (AcP) as donors, respectively (Hentchel and Escalante-Semerena, 2015). This modification has been shown to impact protein structure, protein/DNA-protein interactions and cellular localization, enabling bacteria to quickly adjust to environmental alterations (Carabetta and Cristea, 2017). In addition to lysine acetylation, three new types of lysine acylations have been recently identified by mass spectrometry, including lysine malonylation (Kmal) (Peng et al., 2011), lysine succinylation (Ksucc) (Zhang et al., 2011) and lysine glutarylation (Kglu) (Tan et al., 2014). Due to the presence of an acidic carboxylic group under physiological pH, these newly identified modifications were collectively designated as acidic lysine acyl modifications (Hirschey and Zhao, 2015). Under physiological conditions, acidic modifications alter the charge of lysine residues from +1 to −1, inducing greater changes on protein structure than lysine acetylation when occurring at the same lysine site (Hirschey and Zhao, 2015). Acyl donors, i.e., malonyl-CoA, succinyl-CoA, and glutaryl-CoA, are produced and utilized in the TCA cycle and through the catabolism of amino acids and lipids (Wagner and Hirschey, 2014). The involvement of these acyl-CoAs in metabolism promoted the idea that the corresponding acid lysine modifications might play crucial roles in metabolic regulation. Proteomic studies of these three modifications in eukaryotes revealed several metabolic enzymes were modified, and further pathway enrichment analysis linked acidic acylated substrates and metabolic pathways (Rardin et al., 2013; Weinert et al., 2013; Tan et al., 2014). Up to now, system-wide proteomic characterizations of succinylation was conducted in some bacteria, such as *Escherichia coli* (Colak et al., 2013), *Pseudomonas aeruginosa* (Gaviard et al., 2018), *Staphylococcus aureus* (Tan et al., 2022), *Histoplasma capsulatum* (Xie et al., 2017) etc. In comparison, only a few studies have been reported about lysine malonylation and glutarylation in microorganisms.

Tuberculosis (TB), caused by *Mycobacterium tuberculosis* (*M. tuberculosis*) is considered as one of the most lethal infectious diseases, with its association with HIV/AIDS being particularly serious (Riou and Althaus, 2020). About 1/3 of the annotated *M. tuberculosis* proteome undergoes PTMs, and these proteins play essential functions in the survival of *M. tuberculosis* (Budzik et al., 2020). In *M. tuberculosis*, protein acetylation is a mechanism involved in adaptation to environmental changes, virulence and pathogenesis (Huang et al., 2023). Additionally, *M. tuberculosis* acetyltransferase has the ability to interact with host immune signaling proteins and affect the host’s innate immune response to tuberculosis (Burckhardt and Escalante-Semerena, 2020). Besides lysine succinylation, there are scarce reports on lysine malonylation and glutarylation (Xie et al., 2016; Bi et al., 2022).

In this study, we conducted a comprehensive global characterization of the three acidic lysine acylations in *Mycobacterium smegmatis* (*M. smegmatis*), a non-pathogenic model strain. By using high-affinity antibody enrichment and high-resolution LC-MS/MS, we identified 1,703 malonylated sites, 5,320 succinylated sites, and 269 glutarylated sites. Bioinformatics analysis revealed a significant association between these acidic lysine acylations and the functions of ribosomes, as well as various metabolic pathways. We conducted an in-depth analysis of the shared characteristics of succinylated proteins in *M. smegmatis* and pathogenic *M. tuberculosis*. Besides, we elucidated the influence of lysine succinylation on the functional activity of adenylate kinase and proteasome-associated ATPase. Together, our study represents the first resource on substrate characterization and functional regulation of acidic lysine acylations within *Mycobacterium smegmatis*, offering insights into their interrelation with the biology of infectious pathogens.

## 2 Materials and methods

### 2.1 Cell culture and protein extraction

*Mycobacterium smegmatis* MC2 155 was grown for 60 h in LB medium supplemented with 0.5‰ Tween at 37°C and 220 rpm. The same samples were used for the proteomic analysis of three acidic lysine acylations without the addition of their corresponding short-chain fatty acids. For the proteomic analysis, the cells were centrifuged at 4,000 *g* for 5 min, the supernatant was discarded, and the pellet was collected. The bacterial pellets were washed three times with pre-cooled phosphate-buffered saline (PBS). Cells were lysed on ice for 30 min in a buffer containing 8 M urea in 100 mM NH_4_HCO_3_ (pH 8.0), 1 × Protease inhibitor, 10 mM sodium butyrate and 10 mM nicotinamide. The cell lysates were sonicated for 5 min with 2 s on and 3 s off under 30% power on ice and centrifuged at 21,000 *g* for 10 min at 4°C. The supernatant was transferred to a clean tube and the protein concentration was measured by using the BCA assay.

### 2.2 In-solution digestion

Cell lysates were reduced with 5 mM dithiothreitol under 55°C for 30 min and then alkylated with 15 mM iodoacetamide for 30 min at room temperature in the dark. Then the alkylation was quenched with 30 mM cysteine at room temperature for 30 min. The lysates were diluted with 100 mM NH_4_HCO_3_ to 2 M urea concentration and digested with trypsin at 37°C for 16 h in a trypsin-to-protein ratio of 1:50 (w/w). LysC was then added at LysC-to-protein ratio of 1:100 (w/w) and incubated at 37°C for another 3 h. The peptide samples were desalted by using Sep-Pak vac 1cc (50 mg) C18 cartridges (Waters) and eluted by 75% ACN (0.1% TFA) and finally were dried by SpeedVac.

### 2.3 Enrichment of acylated peptides

Peptide samples were dissolved in ETN buffer (50 mM Tris-HCl, 1 mM EDTA-2Na, 600 mM NaCl, pH 8.0) and centrifuged at 21,000 *g* for 5 min at 4°C. The supernatant was then incubated with acylated antibody conjugated agarose beads (anti-malonyllysine agarose beads: PTM 904, anti-succinyllysine agarose beads: PTM 402, anti-glutaryllysine agarose beads: PTM 1154). Samples were rotated overnight at 4°C after complete mixing. On the next day, the beads were washed by NETN buffer (0.5% NP-40, 50 mM Tris-HCl, 1 mM EDTA-2Na, 600 mM NaCl, pH 8.0) by centrifuging at 500 *g* for 2 min. After that, beads were washed by ETN buffer and HPLC water in turn. Finally, the peptides were eluted twice off the beads with 0.1% trifluoroacetic acid and twice more with an eluent containing 0.1% trifluoroacetic acid in 30% acetonitrile. The enriched peptides were then desalted by ZipTip Pipette Tips (Millipore) and dried by SpeedVac.

### 2.4 LC-MS/MS analysis

The modified peptide samples were dissolved in solvent A (0.1% formic acid and 2% acetonitrile in water, v/v) and loaded into a manually packed reverse-phase C18 column (3 μm particle-sized C18 resin, 75 μm inner diameter × 18 cm length) coupled to an Easy-nLC 1000 system. Peptides were eluted with a 90 min gradient from 1 to 29% solvent B (0.1% formic acid in acetonitrile, v/v) for 74 min, 29 to 45% solvent B for 10 min, 45 to 80% solvent B for 3 min, and 80% solvent B over the last 3 min at a flow rate of 300 nL/min. The eluted peptides were then ionized and analyzed by the Orbitrap Fusion mass spectrometer with a scan range from 300 to 1,400 m/z. The Orbitrap resolution was set as 120,000 at m/z 200 and the maximum injection time was 50 ms. Utilizing higher-energy collisional dissociation (HCD), peptides with 2+, 3+, and 4+ charges were fragmented at a normalized collision energy of 32%. In the ion trap, fragmented ions were detected, with the automatic gain control (AGC) target was set at 7,000 for MS2 analysis.

### 2.5 MS database searching

Raw data analysis was conducted using MaxQuant software (version 2.0.1.0), referencing the UniProt *Mycobacterium smegmatis* database (6,603 proteins, Proteome ID: UP000000757). Trypsin/P was designated as the protease, with the parameter for maximum missed cleavages configured to 2. Modification parameters were configured with carbamidomethyl (C) as a fixed modification, and oxidation (M) and acetyl (Protein N-term) as variable modifications. Besides, malonylation (K) was accounted for in the malonylome, while succinylation (K) and glutarylation (K) were considered for the succinylome and glutarylome data sets, respectively, as a variable modification. The permissible false discovery rate (FDR) for protein, peptide, and modification site identifications was established at 0.01.

### 2.6 Bioinformatics analysis

Sequences identified as contaminants or reverse-identified were excluded, and the modified peptides with a localization probability above 0.75 were retained. Icelogo (version 1.3.8) was used to perform flanking sequence analysis, and the STRING (version 12.0) database was used for the enrichment pathway analysis along with the protein-protein interaction network analysis with a high confidence score (0.7). Cytoscape (3.9.1) software with MCODE plugin tool was used for the connected interaction network clusters analysis.

### 2.7 Constructing plasmids for target genes in wild-type strains

In the mixing system, 1 μL of the PCR target gene fragment, 3 μL of the double digest site (*Eco*RI/*Bam*HI) plasmid vector pET28a, 2 μL of Exnase II, and 4 μL of 5 × CE II buffer was added. The 10 μL reaction mixture was then placed in a 37°C water bath and allowed to stand for 30 min. Subsequently, 5 μL of the mixture was removed to 50 μL of DH5α competent cell and subjected to an ice bath for 30 min. The mixture was then under agitation at 42°C for 30 s and immediately placed back on ice for 3 min. Following this, LB sterile liquid medium was added to resuscitate *Escherichia coli* at 37°C for 1 h. The bacteria were then spread onto Luria Bertani (LB) Plates (Kana, φ9 cm) and incubated for 16 h. After incubation, positive colonies were picked and used to inoculate fresh media, followed by further incubation at 37°C for 16 h. The plasmids were subsequently extracted using a Plasmid mini-extraction kit (TIANGEN-DP103).

### 2.8 Plasmid construction of point mutant strains

Two reaction systems were prepared, 1 μL of wild-type plasmid (100 μg/μL) was added to each system as the PCR template Then, 3 μL of 10 × KOD buffer, 3 μL of dNTP, 1.2 μL of MgSO_4_, and 0.6 μL of KOD DNA polymerase (1.0 U/μL) were added to each system Subsequently, 2 μL of mutant primer-F and 2 μL of mutant primer-R were incorporated into each system. Finally, ddH_2_O was added to a total volume of 30 μL. Two tubes of the PCR products were combined into a single PCR tube, to which an additional 1 μL of KOD enzyme was added for the re-amplification of the target gene. Following this, 1 μL of Dpn 1 enzyme was added and the reaction system was incubated at 37°C for 1 h to digest the template DNA.

### 2.9 Bacterial culture and protein purification

The four overexpression *Mycobacterium smegmatis* strains of MSMEG_1484 (wild-type), MSMEG_1484 (K19E), MSMEG_3902 (wild-type), MSMEG_3902 (K316E) were cultured in Luria Bertani (LB) medium with a final concentration of 0.05 M Kanamycin at 37°C and 220 rpm for 16 h. When the OD_600_ was detected to be 0.4–0.6, the overexpression was induced by adding a final concentration of 0.1 mM isopropyl-β-D-thiogalactoside (IPTG) at 16°C and 160 rpm for 16–20 h. After that, the bacteria were collected by centrifuging at 4°C and 8500 rpm for 5 min, and the supernatant was discarded. The pellet was resuspended in 10 mM Imidazole buffer (PBS) and vortexed thoroughly to ensure complete mixing. Then the resuspended bacteria were sonicated for 5 min and centrifuged at 21,300 *g* for 20 min. The supernatant was collected and passed through a Ni-NTA Agarose Resin column to obtain the target protein.

### 2.10 Enzyme assays

Determination of adenylate kinase and proteasome-associated ATPase activities was performed essentially as previously describe (Kleczkowski and Randall, 1986; De La Mota-Peynado et al., 2013).

The purified adenylate kinase was reacted in the system containing 100 mM Tris-HCl (pH = 7.8), 2 mM ADP, 2 mM MgCl_2_, 0.5 mM NAD^+^, 60 mM KCl, 5 mM D-Glucose, 5 U caproate kinase, and 5 U glucose-6-phosphate dehydrogenase. Then, adenylate kinase activity was determined by measuring the absorbance at 340 nm.

The purified proteasome-associated ATPase was placed in a Tris-HCl (pH = 7.5) reaction system containing 50 mM Tris-HCl buffer (pH = 7.5), 5 mM MgCl_2_, 100 mM NaCl, and 0.5 mM ATP. Then the enzyme activity was determined using the method outlined in the malachite green reagent kit instructions, with absorbance being measured at a wavelength of 630 nm by a spectrophotometer.

### 2.11 Plasmid construction and electrotransformation of *Mycobacterium smegmatis*

Plasmid DNA was extracted from DH5α competent cells using the Beyotime Reagent Kit. Subsequently, the plasmid was introduced into electrocompetent *Mycobacterium smegmatis* cells resuspended in 10% glycerol using an electrotransfer apparatus. After the recombinant plasmids were obtained, they were transformed by electroporation. The recombinant plasmids were mixed with *M. smegmatis* competent cells at 4°C for 5–10 min. The mixture was added to the electric rotor cup, waiting for 2 min, and then electric rotor. Then 500 μL of LB medium containing 0.5‰ Tween 80 was added and incubated for 3–4 h at 37°C and 220 rpm. The bacterial solution was coated with kanamycin resistant solid medium and cultured at 37°C for 2–3 days. Single colonies were obtained the next day, and positive bacteria were cultured in LB medium (0.5‰ Tween 80) at 37°C and 220 rpm.

### 2.12 Flat colony counting and resazurin assay

The glycerol seed of these *Mycobacterium smegmatis* strains were cultured in the LB medium supplemented with 0.5‰ Tween 80. Subsequently, 36 μL of this suspension was combined with kanamycin to achieve a final concentration of 30 μg/mL in 1.8 mL of LB medium supplemented with 0.5‰ Tween 80. Then, 200 μL of this mixture was dispensed into each well of a 96-well plate. The edges were sealed with PBS, and the plate was incubated at 37°C for 24 h. Two independent experiments were conducted for comparing *M.seg*-MSMEG_3902 K316E strain/*M.seg*-MSMEG_3902 strain, and *M.seg*-MSMEG_1484 K19E strain/*M.seg*-MSMEG_1484 strain, respectively.

Each well was gently mixed, and the OD_600*nm*_ was measured. Serial 10-fold dilutions were performed using LB medium with 0.5‰ Tween 80, and 5 μL of each dilution was spread onto Kanamycin-resistant (Kana-R) plates. The plates were inverted and incubated at 37°C for 48 h. Following incubation, colonies were visualized and counted using a fluorescence and chemiluminescence imaging system for flat colony counting. For the remaining *Mycobacterium* cultures in the 96-well plates, 12.5 μg/mL of bladed azure reagent was added. The OD_573*nm*_ was measured after one hour.

### 2.13 Mycobacterial infection

Logarithmically passaged THP-1 cells for three generations were seeded into a 96-well culture plate (150 μL per well), with peripheral wells sealed with 1 × PBS and cultured for 24 h. The cells were then treated with 100 nM PMA to induce adhesion and further incubated for 24 h in fresh 1640 medium with β-Mercaptoethanol, in quintuplicate.

Meanwhile, the *Mycobacterium smegmatis* were pelleted by centrifugation at 4,000 *g*, resuspended in 1640 medium to an OD_600nm_ of 0.1, and equilibrated at 37°C for 30 min. The medium from the THP-1 cells in the 96-well plate was aspirated, and 150 μL of 1640 medium containing equilibrated bacteriophage was added to each well, followed by gentle shaking to ensure thorough mixing. The cells were then incubated at 37°C for 2 h. Subsequently, the medium was removed, and the infected cells were washed with PBS containing 100 μg/mL gentamicin. The cells were further incubated for 24 h in 1640 complete medium supplemented with 25 μg/mL gentamicin.

### 2.14 Cell counting kit-8 assay (CCK-8)

Following infection of THP-1 cells with *Mycobacterium* species, the spent 1640 medium was discarded. Subsequently, 200 μL of CCK-8 assay medium was added to each well according to the manufacturer’s protocol (Biyoungtian CCK-8 Assay Medium Instruction Manual, product code: C0039). The plates were then incubated in a cell culture incubator for 2 h. The absorbance at 450 nm was measured, and the experiment was conducted in triplicate.

### 2.15 Enzyme linked immunosorbent assay (ELISA)

The supernatant from lysed THP-1 cells was harvested, and the expression level of TNF-α was determined by measuring the absorbance at 450 nm, following the protocol outlined in the Absin ELISA Kit instructions (product code: abs51003).

## 3 Results

### 3.1 Landscape of the acidic lysine acylomes in *M. smegmatis*

**FIGURE 1:**
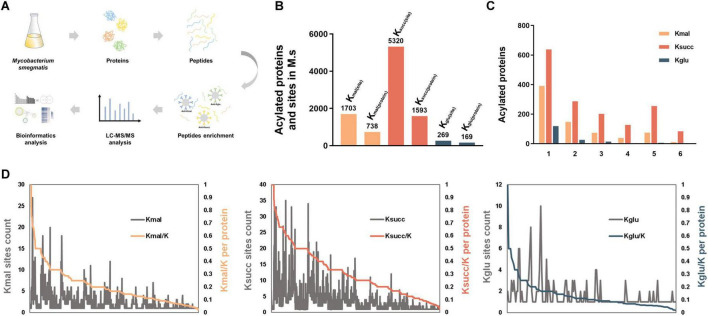
Landscape of acidic lysine acylomes in *M. smegmatis*. **(A)** Experimental procedures for analysis of the three acidic lysine acylations in *Mycobacterium smegmatis*. **(B)** The number of identified lysine acylated proteins and sites in *M. smegmatis*. **(C)** Distribution of lysine acylated sites per protein. **(D)** Proportion of the identified acylated sites in each acylated protein (K-acylated/K).

To systematically investigate the global proteomic characteristics of the three acidic lysine acylations in *M. smegmatis*, we used the high-affinity antibody enrichment combined with high-resolution LC-MS/MS analysis based on the methodologies established in our previous study (Xu et al., 2018; Zhang et al., 2023; Figure 1A). The *M. smegmatis* strain cultured in the same condition was used for exploring the global substrate characterization of the three acidic lysine acylations. In this current study, we totally identified 1,703 lysine malonylated sites on 738 proteins, 5,320 succinylated sites on 1,593 proteins and 269 glutarylated sites on 169 proteins utilizing MaxQuant software, with a scoring threshold exceeding 40 and a localization probability criterion above 75% (Figure 1B and Supplementary Tables 1–3).

Subsequently, we investigated the characteristics of the modified lysine residues and their substrate proteins. The average number of the malonylated, succinylated, glutarylated sites on the corresponding proteins was 2.3, 3.3 and 1.6, respectively. We categorized the modified proteins according to the number of the modified sites they contained. The analysis revealed the distribution of proteins with a single modification site: 391 proteins (53%) in malonylation, 638 proteins (40%) in succinylation and 119 proteins (70%) in glutarylation (Figure 1C). Subsequent analysis of the highly acylated substrates revealed that 84 succinylated proteins exhibited more than 10 modification sites, which is significantly greater compared to the other two types of lysine acylations. Among these proteins, the multifunctional 2-oxoglutarate metabolism enzyme (KGD) and the DNA-directed RNA polymerase (RPOC) subunit beta exhibited the highest degree of succinylation. Additionally, both proteins were highly malonylated. KGD is a key enzyme in the TCA cycle, which regulates the cellular redox status and synthesis of certain amino acids (Bunik, 2019; Mailloux, 2024), and RPOC serves as an essential enzyme in the transcriptional process and regulates gene expression (Hustmyer and Landick, 2024). Furthermore, we found that isocitrate dehydrogenase was the most heavily glutarylated protein (10 sites).

After that, we analyzed the proportion of the identified acylated sites in each substrate protein (Kacylated/K) (Figure 1D, Supplementary Figures 1A, B, C). The results indicated that the extent of lysine malonylation ranged from 2.17% to 100%, with six proteins exhibiting complete malonylation of their lysine residues (Supplementary Table 4A). Concurrently, the small ribosomal subunit protein uS8 and the siderophore binding protein were identified as bearing the highest percentages of lysine succinylation and glutarylation, respectively (Supplementary Tables 4B, C). Specifically, five out of seven lysine residues (71.43%) in the small ribosomal subunit protein uS8 and all lysine residues in the siderophore binding protein were found to be malonylated.

### 3.2 The characterizations of modified substrates and protein-protein interaction networks of the acidic lysine acylomes in *M. smegmatis*

**FIGURE 2:**
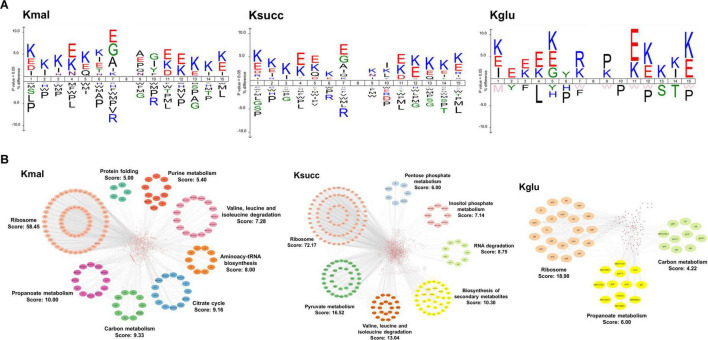
Characterization of modified substrates and protein-protein interaction networks. **(A)** Amino acid preference analysis of sequences flanking the acylated sites. **(B)** The protein-protein interaction networks in malonylated, succinylated, and glutarylated proteins.

We then utilized the icelogo software to conduct flanking sequence analysis on the three acidic acylated lysine sites to explore the amino acid preferences adjacent to the three modified lysine residues. The results indicated a significant overrepresentation of glutamic acid residues at the −1 position in both malonylated and succinylated sites. In contrast, arginine residue was notably overrepresented in the −1 position for the glutarylated sites (Figure 2A). However, the three modifications exhibited distinct amino acid preferences in the +1 position, with alanine, lysine, and proline being significantly overrepresented at malonylated, succinylated, and glutarylated sites, respectively (Figure 2A).

To investigate the potential biological functions of the three acidic lysine acylations, we used the STRING database to conduct the protein-protein interaction network analysis with a high confidence score (0.7). Subsequently, we employed Cytoscape software in conjunction with the MCODE plugin tool to identify highly connected clusters within the interaction network (Figure 2B). We totally identified 735 malonylated proteins, 1,588 succinylated proteins, and 169 glutarylated proteins as nodes. Our findings indicated that the protein cluster associated with ribosomal function exhibited the highest degree cluster among the three acylomes (MCODE score: 58.45 in malonylome, 72.17 in succinylome, and 18.90 in glutarylome). Additionally, pathways related to propanoate metabolism and carbon metabolism were both enriched in the malonylome and glutarylome group. Clusters associated with degradation of valine, leucine, and isoleucine were enriched in the malonylome and succinylome. Furthermore, the findings indicated that the succinylome group encompassed several functionally distinct clusters, such as pyruvate metabolism (MCODE score: 16.52) and biosynthesis of secondary metabolites (MCODE score: 13.04).

### 3.3 Pathway enrichment analysis of the acidic lysine acylomes in *M. smegmatis*

**FIGURE 3:**
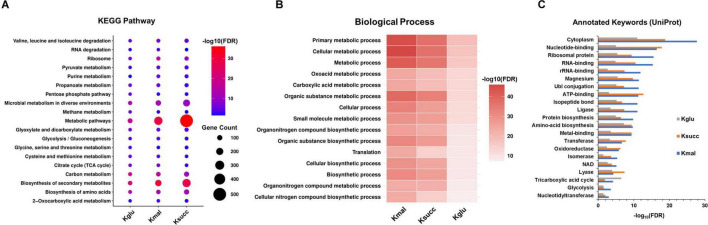
Pathway enrichment analysis of the acidic lysine acylations. **(A)** KEGG pathway analysis. **(B)** Biological process analysis. **(C)** Annotated keywords analysis. FDR < 0.05.

We next performed the pathway enrichment analysis through the KEGG pathway analysis to explore the biological functions associated with the three acidic lysine acylations. Our results indicated that the enrichment pathways of the three modified proteins were predominantly similar, with significant enrichment observed across various metabolic pathways, including TCA cycle, purine metabolism, glycolysis/gluconeogenesis, and ribosome (Figure 3A). Furthermore, our findings revealed that pathways associated with the biosynthesis and metabolism of specific amino acids were exclusively enriched in succinylated proteins, such as alanine, aspartate and glutamate metabolism, arginine biosynthesis, and histidine metabolism. Simultaneously, pathways related to the bacterial secretion system, nucleotide excision repair, and protein export exhibited specific enrichment for lysine-malonylated proteins (Supplementary Figure 1).

We conducted an in-depth analysis of biological processes to elucidate the characteristics of the three acidic lysine acylations (Figure 3B). Our findings indicated that proteins subjected to these acylations are predominantly enriched in cellular metabolic processes, primary metabolic processes, and organic substance metabolic processes. Additionally, regulation of translation and glyoxylate metabolic process were found to be uniquely enriched in lysine malonylated and glutarylated proteins. After that, the annotated keyword analysis revealed that certain enzyme-related functions including isomerase, oxidoreductase, transferase, and ligase were enriched in all the three acylations (Figure 3C).

### 3.4 Comprehensive analysis of the of the three acylated common substrates

**FIGURE 4:**
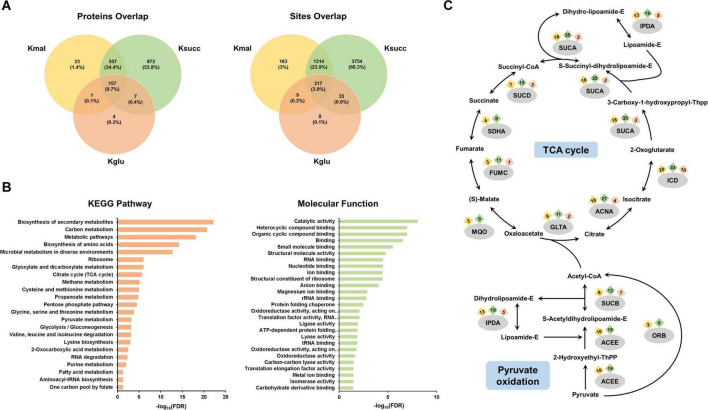
Systematic analysis of the common substrates. **(A)** Venn diagram of the three acidic lysine acylations. **(B)** Pathway enrichment analysis of the 157 shared acylated proteins. **(C)** Occurrence of three acylations in enzymes within the TCA cycle and pyruvate oxidation pathway.

To thoroughly investigate the common characteristics of the three acylated substrates, we conducted an analysis of the common modified proteins and lysine residues associated with the three types of acidic lysine acylations. Our data indicated that a total of 157 modified proteins (9.7%) and 217 lysine residues (3.9%) were shared among the three acylation modifications in *M. smegmatis* (Figure 4A).

Subsequently, we conducted the pathway enrichment analysis on these 157 common modified proteins to elucidate shared characteristics or functions potentially associated with the three modifications. We discovered that they were enriched in important biological processes, including ribosome, TCA cycle, glyoxylate and dicarboxylate metabolism, and glycolysis/gluconeogenesis (Figure 4B). In addition, the molecular function analysis indicated that functions associated with gene expression, such as translation factor activity, RNA binding, and nucleotide binding, were found to be enriched (Figure 4B). The Annotated Keyword analysis demonstrated similar enrichment results (Supplementary Figure 2A). We further performed the protein-protein interaction network analysis of the 157 shared proteins. The results showed three highly enriched protein clusters, including ribosome (MCODE score: 18.900), carbon metabolism (MCODE score: 7.636), and pentose phosphate pathway (MCODE score: 5.091) (Supplementary Figure 2B). We then analyzed the common proteins enriched in TCA cycle and pyruvate oxidation pathways, and our results showed that nearly all enzymes in these two central metabolic pathways possess multiple distinct acylation sites (Figure 4C).

After that, we investigated the common modified lysine residues shared by the three acidic lysine acylations. Our results demonstrated that isocitrate dehydrogenase, which plays a role in TCA cycle, had eight shared acylated sites. In addition, several lysine residues on chaperonin GroEL 2, elongation factor Tu, ketol-acid reductoisomerase were commonly modified by all three lysine modifications (Supplementary Figure 2C). For these two proteins, chaperonin GroEL is a molecular chaperone that mediates protein folding (Krishna et al., 2007), and elongation factor Tu is a G protein that catalyzes the association of ribosomes with aminoacyl-tRNA (Harvey et al., 2019). The results suggested that the three acidic lysine acylations may mediate important biological processes such as cellular metabolism and protein synthesis.

### 3.5 The common characteristics of succinylated proteins in *M. smegmatis* and *M. tuberculosis*

**FIGURE 5:**
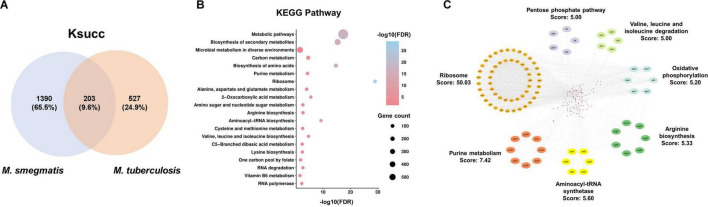
Characteristics of common succinylated proteins in *M. smegmatis* and *M. tuberculosis*. **(A)** 203 common succinylated proteins. **(B)** KEGG pathway enrichment analysis of common substrates. FDR < 0.05. **(C)** Protein-protein interaction network analysis by the STRING database with a high confidence score (0.7).

We next investigated the common characteristics of succinylated proteins shared by *M. smegmatis* and *M. tuberculosis* through integrating our data with the data available in the CPLM database (Supplementary Table 5). This analysis identified a total of 203 proteins that were common between the 1,593 lysine succinylated proteins of *M. smegmatis* and the 730 lysine succinylated proteins of *M. tuberculosis* (Figure 5A). Subsequently, we conducted a KEGG pathway analysis on the 203 substrate proteins. The results revealed significant enrichment in ribosome and metabolic pathways. Additionally, several biosynthetic processes, including the biosynthesis of secondary metabolites, amino acids, and aminoacyl-tRNA, were found to be highly enriched. We observed a significant enrichment in pathways associated with amino acid synthesis and metabolism, such as valine, leucine and isoleucine biosynthesis and alanine, aspartate and glutamate metabolism (Figure 5B). This result suggested that extensive lysine succinylation modification may promote amino acid synthesis in mycobacteria. Subsequently, we conducted a protein-protein interaction network analysis utilizing the STRING database with a high confidence threshold of 0.7. We then employed Cytoscape software with MCODE plugin tool to identify highly connected interaction network clusters (score ≥ 5). Similarly, our result indicated that the protein cluster involved in ribosomal function was highly enriched (MCODE score: 50.03). Meanwhile, purine metabolism (score: 7.42) and aminoacyl-tRNA synthetase (score: 5.60) represented the other significantly enriched clusters within the group of overlapping succinylated proteins (Figure 5C).

### 3.6 Functional exploration of the modified lysine residues in adenylate kinase and proteasome-associated ATPase

**FIGURE 6:**
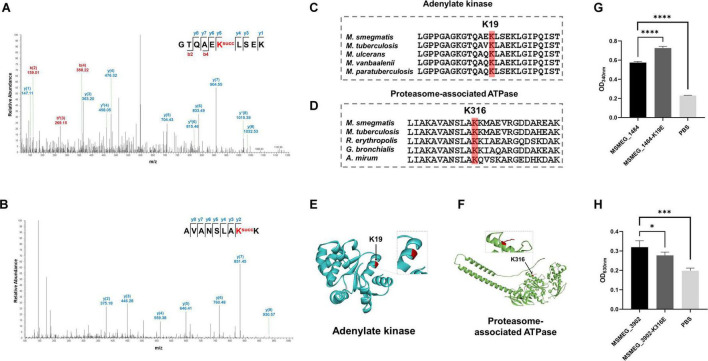
Relative enzymatic activity of adenylate kinase and proteasome-associated ATPase at site-specific level. **(A,B)** MS/MS spectra of the succinylated peptides showing the modified K19 of adenylate kinase (MSMEG_1804) and the modified K316 of proteasome-associated ATPase (MSMEG_3902). **(C,D)** Sequence alignment of adenylate kinase and proteasome-associated ATPase from different species. **(E,F)** Visualization of the succinylation site in the constructed crystal structure of adenylate kinase and proteasome-associated ATPase. **(G,H)** Relative absorbance of the two substrates with their mutants after enzyme activity reaction (*P-value < 0.05; ***P-value < 0.001; ****P-value < 0.0001, Student’s *t*-test).

Lysine acylation plays a role in regulating protein function, controlling diverse signaling and regulatory pathways (Shen et al., 2023). In our proteomic data, the K19 of adenylate kinase (MSMEG_1484) (score: 80.68) and the K316 of proteasome-associated ATPase (MSMEG_3902) (score: 137.18) were identified to be succinylated in *M. smegmatis* (Figures 6A,B). The adenylate kinase facilitates the reversible phosphoryl transfer between ATP and AMP, which is crucial for cellular energy equilibrium and adenine nucleotide homeostasis (Ren et al., 2005; Fujisawa, 2023), and the ATPase plays a pivotal role in the proteasomal degradation process (Zhang and Mao, 2020). The sequence conservation analysis revealed that lysine residues K19 (MSMEG_1484) and K316 (MSMEG_3902) were highly conserved across different species (Figures 6C–F). To investigate the impact of lysine succinylation on these two enzymes, we constructed mutant proteins as negative controls by substituting lysine at position K19 in MSMEG_1484 and lysine at position K316 in MSMEG_3902 with glutamate, thereby mimicking a succinylated lysine state. The *in vitro* enzymatic activity assays demonstrated that MSMEG_1484-K19E exhibited increased enzymatic activity relative to wild-type MSMEG_1484, whereas the MSMEG_3902-K316E showed decreased enzymatic activity compared to wild-type MSMEG_3902 (Figures 6G, H). The results indicated that lysine succinylation could regulate the enzymatic activity of adenylate kinase and proteasome-associated ATPase in mycobacteria.

### 3.7 Regulatory roles of lysine acylation in mycobacterial infection

**FIGURE 7:**
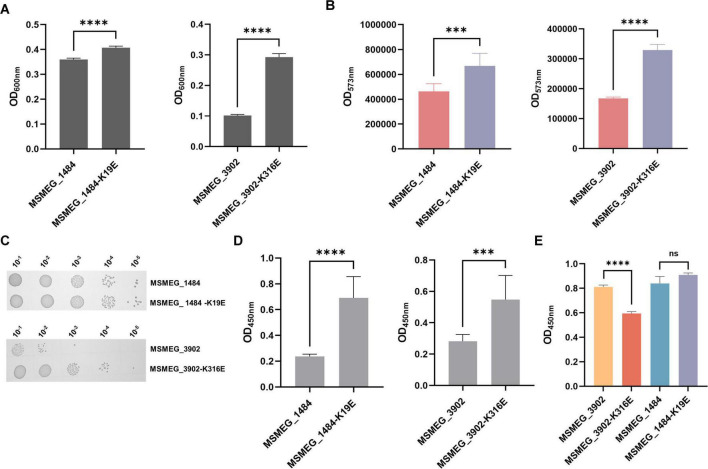
Impact of adenylate kinase and proteasome-associated ATPase succinylation on mycobacterial physiology and infection process. **(A)** Absorbance at 600 nm, indicative of cell growth, after 24 h of culturing *Mycobacterium smegmatis* overexpressing MSMEG_1804, MSMEG_1804-K19E, MSMEG_3902, and MSMEG_3902-K316E (****P-value < 0.0001, Student’s *t*-test). **(B)** Absorbance at 573 nm, which serves as a measure of cell viability, as indicated by the resazurin reduction assay (***P-value < 0.001; ****P-value < 0.0001, Student’s *t*-test). **(C)** Colony-forming units of *Mycobacterium smegmatis* on kanamycin-resistant (Kana) agar plates. **(D)** Cell viability measured by the absorbance at 450 nm using the CCK-8 assay after MSMEG_1804, MSMEG_1804-K19E, MSMEG_3902, and MSMEG_3902-K316E infection THP-1 (***P-value < 0.001; ****P-value < 0.0001, Student’s *t*-test). **(E)** The TNF-α level as measured by the absorbance at 450 nm in cell supernatants by ELISA (****P-value < 0.0001; ns, *P*-value > 0.05, Student’s *t*-test).

Next, we further explored whether lysine succinylation of adenylate kinase and proteasome-associated ATPase could affect mycobacterial physiological characteristics and infection process. We constructed the *M.seg* strains with MSMEG_1484 (wild-type), MSMEG_1484 K19E, MSMEG_3902 (wild-type), MSMEG_3902 K316E overexpression, and then evaluated the impact of wild-type and mutant enzymes on mycobacterial physiological and pathogenic processes. Based on the growth experiments, we observed that the *M.seg*-MSMEG_1484 K19E or *M.seg*-MSMEG_3902 K316E mutant strains exhibited improved growth and cellular activity compared to their corresponding wild-type strains, respectively (Figures 7A, B, C). In addition, the survival rate of THP-1 cell was elevated in the infected groups of two mutant *M.seg* strains than in their wild-type *M.seg* group (Figure 7D). Furthermore, ELISA analysis revealed a reduction in TNF-α secretion by THP-1 cells infected with *M.seg*-MSMEG_3902 K316E strain in comparison with that observed in its wild-type strain (Figure 7E). This result indicated that K316 is a key residue in proteasome-associated ATPase, impacting the growth and pathogenicity of *Mycobacterium smegmatis*.

## 4 Discussion

In our study, we characterized the first and largest acidic lysine acylomes in Mycobacteria, with the identification of 1,703 malonylated sites, 5,320 succinylated sites, and 269 glutarylated sites in *M. smegmatis* proteins. The flanking sequence analysis of the acidic acylated lysine sites revealed distinct amino acid preferences adjacent to the modified lysine residues. Specifically, there was a notable preference for glutamate in the malonylome and succinylome, whereas such a preference was not observed in the glutarylome. Subsequent analysis of protein-protein interaction networks and pathway enrichment analysis revealed a significant association with ribosome functions and metabolic pathways. Besides, we analyzed the 157 modified proteins that are shared among the three modifications to comprehensively explore the common characteristics of the acidic acylations. We identified an enrichment in essential biological processes within organisms, including ribosome function, the TCA cycle, glyoxylate and dicarboxylate metabolism, and glycolysis/gluconeogenesis. Meanwhile, the exploration of the common succinylated proteins shared by *M. tuberculosis* and *M. smegmatis* showed deep lysine succinylation modification may contribute to amino acid synthesis in mycobacteria. Furthermore, we elucidated the influence of lysine succinylation on the functional activity of adenylate kinase and proteasome-associated ATPase. In summary, our study elucidated the extensive range of acidic acylation substrates in *Mycobacterium smegmatis*, thereby offering a resource for further biological functional research of acidic lysine acylations in *Mycobacterium smegmatis*.

Pathogenic mycobacteria are noted for their capacity to interfere with the host’s energy metabolism, a mechanism that frequently results in severe diseases within host (Ehrt et al., 2018). For instance, the Rv1813c protein of *M. tuberculosis* modulates host mitochondrial function by enhancing ATP production and delaying the release of cytochrome c, which may contribute to the pathogen’s persistence and evasion of the host immune response (Martin et al., 2023). Our research has identified a significant enrichment of three acidic lysine acylation modifications within *Mycobacterium smegmatis*, including the succinylation, malonylation, and glutarylation. These alterations are particularly evident in vital metabolic pathways, such as the tricarboxylic acid (TCA) cycle and the pyruvate oxidation process, both of which are fundamental for energy production and metabolic regulation. The observed enrichment of these acylation modifications may reflect a complex mechanism through which mycobacteria adapt to the host cellular environment. Through these modifications to regulate key metabolic enzymes, mycobacteria may potentially manipulate the host’s metabolic environment to sustain their own survival and fulfill their energy requirements. This strategic adaptation may confer mycobacteria a competitive advantage to mycobacteria within the host, potentially enabling them to circumvent immune defenses and establish a persistent infection. Our findings not only enhance our understanding of the intricate relationship between mycobacterial acylation and host-pathogen interactions but also imply the potential utility of these acidic acylations as biomarkers or therapeutic targets. Currently, a variety of post-translational modification (PTM) regulatory enzymes have been identified as potential targets for drug development, including the protein kinase PKnG, the ubiquitin-like protein ligase PafA, and the acetyltransferase Eis. The disruption of the protein kinase PknG significantly increases the susceptibility of mycobacteria to antibiotics and concurrently diminishes their drug resistance (Khan et al., 2018). Moreover, the application of PafA inhibitors has been observed to suppress the proliferation of the attenuated *Mycobacterium tuberculosis* strain H37Ra (Li et al., 2022). Additionally, Eis inhibitors have been shown to reinstate the efficacy of kanamycin in strains of *Mycobacterium tuberculosis* that exhibit resistance to this antibiotic (Pang et al., 2022). Therefore, in-depth analysis of the mycobacterial substrate profile not only facilitates the identification of novel therapeutic targets for tuberculosis, but also holds promise for mitigating the emergence of drug-resistant strains. In our subsequent research, we intend to conduct a comparative analysis of the quantitative variations in post-translational modifications (PTMs) between drug-sensitive and drug-resistant strains, aiming to identify potential modification sites that may act as biomarkers for drug sensitivity.

In addition to the lysine succinylation, malonylation, and glutarylation studied in this paper, other acidic lysine acylation modifications exist, such as lysine methylmalonylation, a modification that has been discovered in humans and mice. This modification is recognized for its ability to inhibit the activity of enzymes involved in the urea cycle and glycine cleavage pathway, thereby contributing significantly to the pathogenesis of methylmalonic acidemia (Head et al., 2022). Despite its recognized importance in eukaryotic systems, the study of lysine methylmalonylation has not been extended to prokaryotes, resulting in a gap in our understanding of its potential impact on these organisms. Future research should investigate the prevalence and functional implications of lysine methylmalonylation, as well as analogous acidic acylation modifications, within prokaryotic organisms. Examining these acidic acylation modifications could elucidate the complex regulatory mechanisms of bacterial metabolic pathways and the intricate relationships between hosts and microbes. Understanding these modifications in prokaryotes has the potential to provide novel insights into the treatment of infections caused by pathogenic bacteria.

## Data Availability

The datasets presented in this study can be found in online repositories. The names of the repository/repositories and accession number(s) can be found in this article/Supplementary material.
