# Supplementary material for: Characterization of acidic lysine acylations in mycobacteria
Source: Front Microbiol. 2024 Dec 10;15:1503184. doi: 10.3389/fmicb.2024.1503184 (PMC11667787; doi:10.3389/fmicb.2024.1503184)
Supplement: Supplementary file 6 [file Data_Sheet_1.docx]

***Supplementary Material***

**Characterization of Acidic Lysine Acylations in Mycobacteria**

Tong Ye^1,2^#, Danfeng Wang^3,4^#, Yewen Sun^4^#, Shuyu Xie^1,2^, Tianqi Liu^4^, Nana Tian^3,4^, Minjia Tan^1,2,3,4^*, Jun-Yu Xu^1,2,3,4^*.

^1^School of Chinese Materia Medica, Nanjing University of Chinese Medicine, Nanjing 210023, Jiangsu, China.

^2^State Key Laboratory of Drug Research, Shanghai Institute of Materia Medica, Chinese Academy of Sciences, Shanghai, 201203, China

^3^School of Pharmacy, Zunyi Medical University, Zhuhai, 519041, China.

^4^Zhongshan Institute for Drug Discovery, Shanghai Institute of Materia Medica, Chinese Academy of Sciences, Guangdong, China.

# These authors contributed equally.

*To whom correspondence should be addressed:

Jun-Yu Xu, Email: [jyxu@simm.ac.cn](mailto:jyxu@simm.ac.cn)

Minjia Tan, Email: mjtan@simm.ac.cn


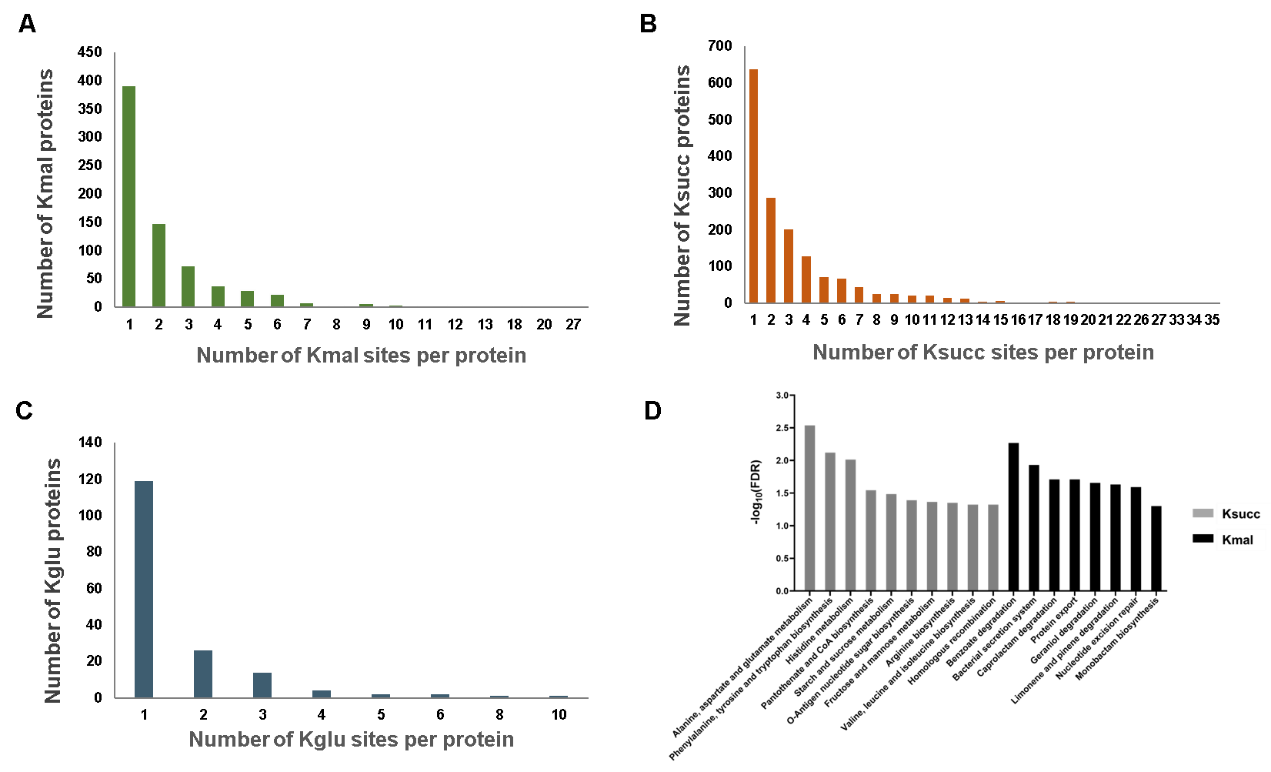


Figure S1. (A)(B)(C) Number of Kmal/Ksucc/Kglu sites per protein. (D) The specific enriched pathways in the malonylated and succinylated proteins using KEGG pathways assay by the STRING database. FDR < 0.05.


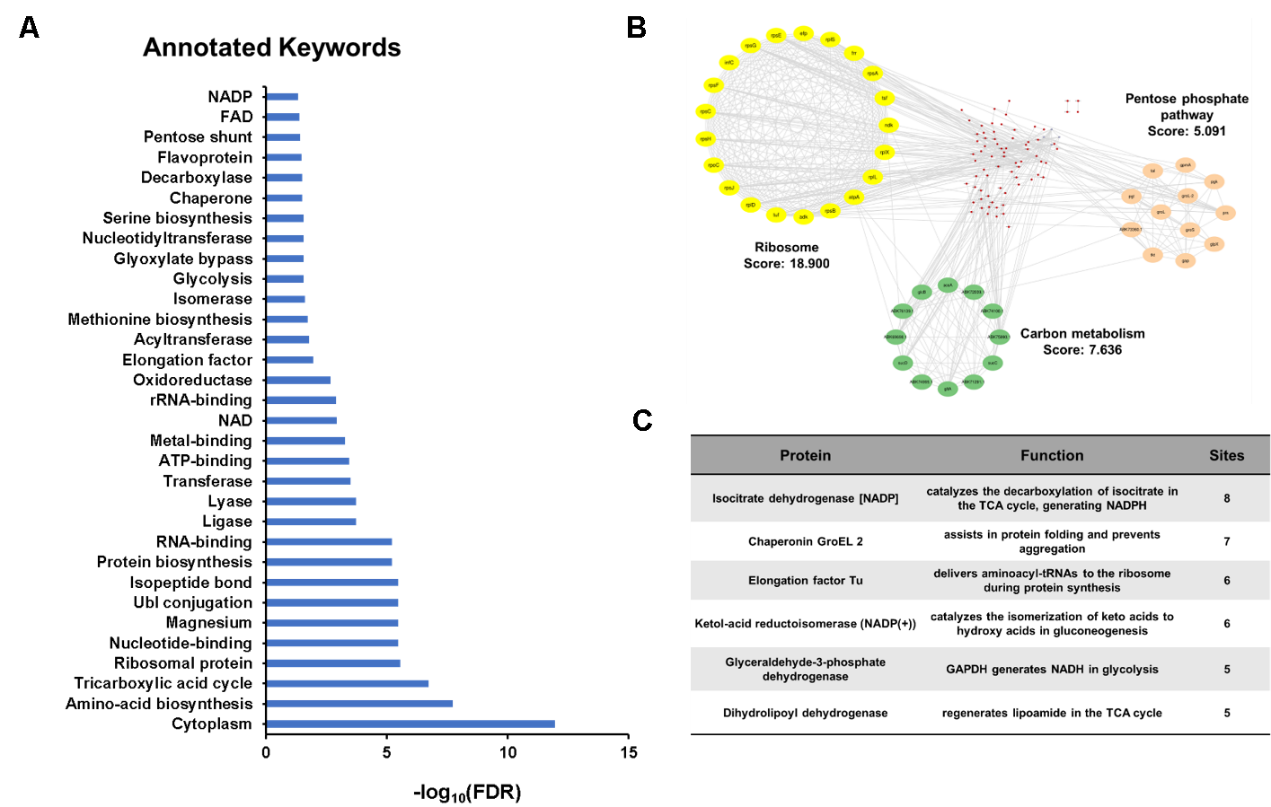


Figure S2. (A) Annotation keywords of the 157 shared acylated proteins in *Mycobacterium smegmatis*. (B) Protein-protein interaction networks analysis in common acylated proteins. (C) List of proteins with multiple modified lysine residues shared by the three acidic lysine acylations.

**Supplementary table**

Table S1. List of malonylated proteins identified in *Mycobacterium smegmatis*.

Table S2. List of succinylated proteins identified in *Mycobacterium smegmatis*.

Table S3. List of glutarylated proteins identified in *Mycobacterium smegmatis*.

Table S4. The modified lysine sites and ratio per protein.

Table S5. List of overlap Ksucc proteins in *Mycobacterium smegmatis* from our dataset and *Mycobacterium tuberculosis* from CPLM database.

**Data availability statement**

The mass spectrometry proteomics data have been deposited to the ProteomeXchange Consortium (https://proteomecentral.proteomexchange.org) via the iProX partner repository with the dataset identifier PXD056151.
